# Genome-wide association study overcomes the genome complexity in autohexaploid chrysanthemum and tags SNP markers onto the flower color genes

**DOI:** 10.1038/s41598-019-50028-z

**Published:** 2019-09-26

**Authors:** Katsuhiko Sumitomo, Kenta Shirasawa, Sachiko Isobe, Hideki Hirakawa, Tamotsu Hisamatsu, Yoshihiro Nakano, Masafumi Yagi, Akemi Ohmiya

**Affiliations:** 1 Institute of Vegetable and Floriculture Sciences, NARO, Tsukuba, Ibaraki 305-0852 Japan; 2 Kazusa DNA Research Institute, Kisarazu, Chiba 292-0818 Japan

**Keywords:** Polyploidy in plants, Plant molecular biology, Plant breeding

## Abstract

The use of DNA markers has revolutionized selection in crop breeding by linkage mapping and QTL analysis, but major problems still remain for polyploid species where marker-assisted selection lags behind the situation in diploids because of its high genome complexity. To overcome the complex genetic mode in the polyploids, we investigated the development of a strategy of genome-wide association study (GWAS) using single-dose SNPs, which simplify the segregation patterns associated polyploids, with respect to the development of DNA markers. In addition, we employed biparental populations for the GWAS, wherein the SNP allele frequency could be predicted. The research investigated whether the method could be used to effectively develop DNA markers for petal color in autohexaploid chrysanthemum (*Chrysanthemum morifolium*; 2n = 6x = 54). The causal gene for this trait is already-known *CmCCD4a* encoding a dioxygenase which cleaves carotenoids in petals. We selected 9,219 single-dose SNPs, out of total 52,489 SNPs identified by dd-RAD-Seq, showing simplex (1 × 0) and double-simplex (1 × 1) inheritance pattern according to alternative allele frequency with respect to the SNP loci in the F_1_ population. GWAS, using these single-dose SNPs, discovered highly reproducible SNP markers tightly linked to the causal genes. This is the first report of a straightforward GWAS-based marker developing system for use in autohexaploid species.

## Introduction

Polyploidy is the condition in which multiple sets of chromosomes are present in a single nucleus. Polyploid plants are classified into the two major categories, namely, autopolyploids (involving only one genome) and allopolyploids (involving two or more different sub-genomes). In bivalent formation at meiotic prophase I in allopolyploids, preferential chromosome pairing between homologous chromosomes generally occurs by prevention of homeolog pairing. Thus, the manner of inheritance is diploid-like in allopolyploids, i.e., Mendelian inheritance. In contrast, random chromosome pairing primarily occurs during bivalent formation in autopolyploids, with a few exceptions^1,2^. For autohexaploids (6x), for example, one chromosome randomly pairs with one of the other five homologous chromosomes, resulting in a complex inheritance pattern.

Autopolyploid plant species are important in plant evolution and as crops, but the use of DNA marker-assisted breeding in autopolyploids still lags behind that of diploid crops because linkage mapping and QTL analysis for the development of DNA markers in autopolyploids needs specialized methods. The complicated segregation patterns of markers in autopolyploids requires the use of specific statistical methods for the estimation of recombination frequency to achieve QTL detection^3^. Progress has been made for the development of DNA markers in autotetraploid crops (e.g., potato)^4^, and the dedicated program is available for linkage mapping^5^. Recently, much progress has been reported with respect to linkage mapping in autohexaploid crops, including sweet potato (*Ipomoea batatas*)^6–8^ and cultivated chrysanthemum (*Chrysanthemum morifolium*)^9^. QTL detection has been achieved in chrysanthemum using highly sophisticated methods that handles genome complexity^9^. However, the issue of genome complexity remains to be resolved and circumvented in autohexaploids.

To simplify the complex genetic mode in the autohexaploids, Shirasawa *et al*.^8^ used only simplex SNPs, which are presented in only one of the six homologous chromosomes, for genetic analysis in the hexaploids. The simplex SNPs are expected to segregate in progeny in accordance with the simple Mendelian genetic law. Indeed, Shirasawa *et al*.^8^ have employed the simplex SNPs to establish a genetic map in sweetpotato, which consisted of roughly 15 pairs of 6 homologous linkage maps corresponding to the number of the chromosomes (2n = 6x = 90). Therefore, we hypothesized that the simplex SNPs could be used for association analysis to capture simplex genetic loci controlling phenotypes of interest.

To evaluate this method, we used cultivated chrysanthemum as the material in this study. It is an allogamous autohexaploid plant with 54 chromosomes (2n = 6x = 54)^10–13^, which is used worldwide for ornamental and medicinal purposes. The white color of petals (of ray florets) is dominant over yellow in chrysanthemums^14^, and allelic variation at a single genetic locus determines whether the petal becomes white or yellow, with carotenoid pigmentation in the petal being controlled by the dominant allele of an inhibitor^11^. When a chrysanthemum plant possesses the dominant allele, the carotenoid concentration in petals is very low and its flower color shows white or pink depending on the absence or presence of anthocyanin pigment, respectively. Conversely, the nulliplex condition for the dominant allele (where none of the six copies of the gene are dominant) gives yellow or bronze petals by accumulating carotenoids. A gene encoding a carotenoid cleavage dioxygenase (*CmCCD4a*) has been identified as the single dominant gene that causes the inhibition of carotenoid accumulation in white petals in chrysanthemum^15^. In the present study, we tested whether the genome-wide association study (GWAS) method could unravel the genome complexity in autohexaploid chrysanthemum to enable the development of DNA markers for carotenoid pigmentation in petals, and the accuracy of the strategy was tested to check whether the DNA marker correctly located this flower color gene.

## Results

### Inheritance of carotenoid cleavage and the causal gene *CmCCD4a*

**Figure 1:**
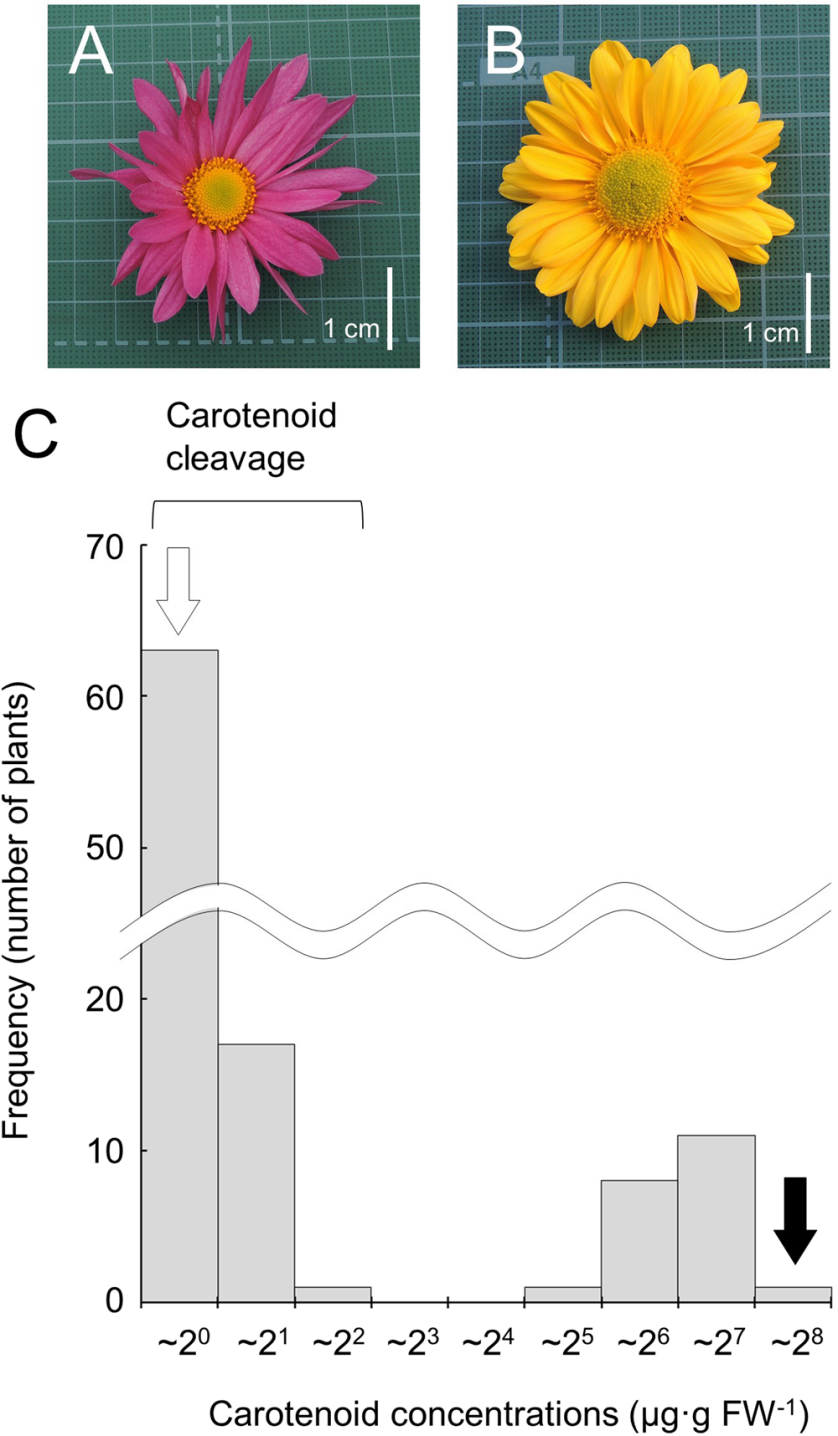
Flowers of the parents and carotenoid concentrations of the F_1_ population. (**A**,**B**) Flowers of ‘Ariesu’ (**A**; pink petals, due to carotenoid cleavage but the presence of anthocyanin pigment) and ‘Yellow Queen’ (**B**). (**C**) Frequency distribution of carotenoid concentrations of the F_1_ individuals and their parents ‘Ariesu’ (white arrow) and ‘Yellow Queen’ (black arrow).

Carotenoid concentrations in petals of ray florets of ‘Ariesu’ (pink petals, due to carotenoid cleavage but the presence of anthocyanin pigment) and ‘Yellow Queen’ were 0.7 and 216.9 μg·g^−1^ fresh weight (FW), respectively (Fig. 1). Phenotypic segregation was observed in the F_1_ population from reciprocal crosses between the two cultivars. Eighty-one individuals from the pooled F_1_ population (n = 102) contained < 2.2 μg·g^−1^ FW as low carotenoid concentrations inherited from ‘Ariesu’ and were scored as exhibiting carotenoid cleavage, whereas 21 contained >29.6 μg·g^−1^ FW, exhibiting high carotenoid concentrations inherited from ‘Yellow Queen’. Segregation data for carotenoid cleavage in the F_1_ population gave an extremely close fit to a 4:1 ratio (carotenoid cleavage^+^:carotenoid cleavage^−^ = 81:21; χ^2^ = 0.022, *P* = 0.88) of segregation from the duplex × nulliplex (AAaaaa × aaaaaa) cross in the hexasomic inheritance model, suggesting that ‘Ariesu’ possessed two dominant alleles at the genetic locus for carotenoid cleavage.

*CmCCD4a* is the causal gene of carotenoid cleavage in petals of ray florets of chrysanthemums. *CmCCD4a* comprises a small gene family in the chrysanthemum genome^16,17^. Products for *CmCCD4a* were amplified by PCR only in ‘Ariesu’, using primers designed in the conserved region of *CmCCD4a* homologs; in contrast, no band was observed in ‘Yellow Queen’ (see Supplementary Fig. S1). Subsequent sequencing analysis revealed that at least three *CmCCD4a* homologs were present in the ‘Ariesu’ genome, which were identical to *CmCCD4a-1* (DDBJ accession number: AB627797), *CmCCD4a-4* (AB627802) and *CmCCD4a-*5 (AB695091) as reported in previous studies^16,17^. While both *CmCCD4a-1* and *CmCCD4a-5* had complete open reading frames, *CmCCD4a-4* was identified to be a pseudogene because stop codons were found in the protein-coding sequence^17^. Therefore, ‘Ariesu’ appeared to carry two functional *CmCCD4a*.

Each *CmCCD4a* derived from ‘Ariesu’ was analyzed using allele-specific PCR (ASP-PCR). F_1_ individuals having functional *CmCCD4a-1* and/or *CmCCD4a-5* showed carotenoid cleavage in the petals of ray florets (see Supplementary Table S1). Conversely, F_1_ individuals lacking functional *CmCCD4a* accumulated high concentrations of carotenoids in their petals of ray florets. The presence or absence of functional *CmCCD4a* was perfectly matched to carotenoid cleavage/non-cleavage in the F_1_ population, clearly confirming that *CmCCD4a* is the single dominant gene responsible for carotenoid cleavage in petals of ray florets, resulting in white or pink petals.

The observed segregation ratio (presence:absence) for the inheritance of three *CmCCD4a* homologs in the F_1_ population was 54:48 for *CmCCD4a-1*, 54:48 for *CmCCD4a-4* and 51:51 for *CmCCD4a-5* (Table 1). Each ratio closely fitted a 1:1 ratio, the expected segregation for simplex × nulliplex (1 × 0) cross for hexasomic inheritance. When a genotype was defined according to the combination of the presence or absence of the three genes in a plant, the numbers of individuals having each genotype in the F_1_ population are shown in Table 2. When the genotypes of the two functional genes *CmCCD4a-1* and *CmCCD4a-5* were combined, the segregation ratio (both *CmCCD4a-1* and *CmCCD4a*-*5*:only *CmCCD4a-1*:only *CmCCD4a-5*:null = 24:30:27:21) showed a good fit to a 2:3:3:2 ratio (χ^2^ = 1.09, *P* = 0.78) for a duplex × nulliplex cross. This suggests that two functional *CmCCD4a* are located in a homologous chromosome group and correspond to two dominant alleles at a genetic locus for carotenoid cleavage in ‘Ariesu’. The genotype of *CmCCD4a-1* was always associated with that of the pseudogene *CmCCD4a-4* in F_1_ individuals, showing that these genes are tightly linked.

**Table 1:** Segregation of three *CmCCD4a* homologs in the F_1_ population.

Gene	Number of F_1_ plants	
	Presence	Absence
*CmCCD4a-1*	54	48
*CmCCD4a-4*	54	48
*CmCCD4a-5*	51	51

**Table 2:** Combined genotypes of three *CmCCD4a* homologs and number of individuals in the F_1_ population.

*CmCCD4a*-*1/4/5*	Number of F_1_ plants
+/+/+	24
+/+/−	30
+/−/+	0
−/+/+	0
+/−/−	0
−/+/−	0
−/−/+	27
−/−/−	21

^+^Presence.

^−^Absence.

### GWAS for carotenoid concentrations

Approximately 1.2 M high-quality reads per sample were obtained from the F_1_ population (n = 102), and 82.3% of them were mapped onto the reference sequence of the *C*. *seticuspe* genome, CSE_r1.0^18^. A total of 52,489 high-confidence single-nucleotide polymorphism (SNP) candidates were identified. The alternative allele frequency (AAF) scores were distributed as shown in Supplementary Fig. S2. From these, 5,509 double-simplex (Aaaaaa × Aaaaaa or AAAAAa × AAAAAa) and 3,710 simplex (Aaaaaa × aaaaaa, aaaaaa × Aaaaaa, AAAAAa × AAAAAA, or AAAAAA × AAAAAa) SNPs significantly fitting to the expected ratio of 3:1 and 1:1, respectively, were selected in accordance with the AAF scores and chi-squared tests (*P* ≥ 0.01) as mentioned in “Methods”

**Figure 2:**
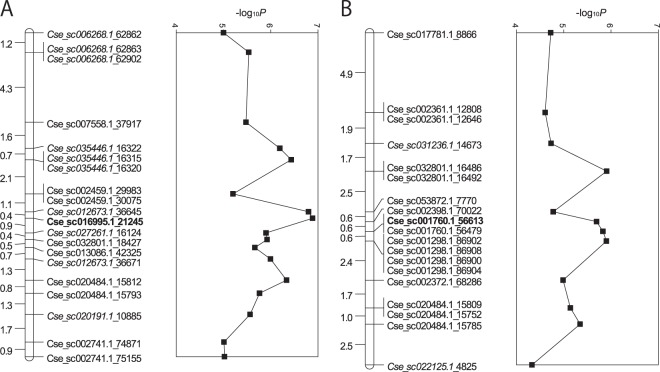
Linkage maps with the 39 simplex single-nucleotide polymorphisms associated with carotenoid concentrations in petals of ray florets in ‘Ariesu’ by genome-wide association study. Two linkage groups (**A**,**B**) are corresponding to two homoeologous chromosomes of the hexaploid genome of *C*. *morifolium*. SNP loci are indicated on right side of the linkage maps. SNPs in italic are located on LG8 of *C*. *seticuspe* linkage maps^18^, and those in bold are validated by ASP-PCR. The −log_10_*P* values for each locus from genome-wide association study are shown by line graphs.

Because no population structure was observed in the F_1_ populations (see Supplementary Fig. S3), initially, we used general linear modeling (GLM) for GWAS. The GLM analysis with the 9,219 (i.e., 5,509 + 3,710) SNP markers identified that 46 SNP markers (39 simplex and 7 double-simplex SNPs) were significantly associated with carotenoid concentrations in petals of ray florets. To clarify the number of genetic loci involved, linkage analysis was performed with the 39 simplex SNPs observed in ‘Ariesu’ to generate two linkage groups (Fig. 2). Among the 21 contigs, on which the 39 SNPs were detected, six sequences (Cse_sc006268.1, Cse_sc012673.1, Cse_sc022125.1, Cse_sc027261.1, Cse_sc031236.1 and Cse_sc035446.1) were found on the linkage group (LG) 8 of the *C*. *seticuspe* linkage maps^18^ while the others were not assigned on the map except for one on LG 2 (Cse_sc020191.1). The result of GLM was supported by mixed linear model (data not shown), in which only the kinship relationship of the materials was used.

**Figure 3:**
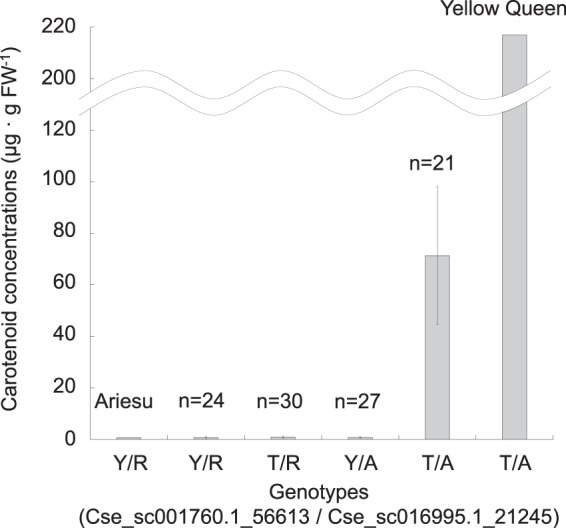
Mean values for carotenoid concentrations with different combination of two single-nucleotide polymorphisms in the F_1_ individuals and their parents ‘Ariesu’ and ‘Yellow Queen’. n: number of F_1_ plants. Error bars indicate standard deviation. The Y and R genotype show degenerate base of heterozygote of T/C on Cse_sc001760.1_56613 and A/G on Cse_sc016995.1_21245, respectively.

To validate the GWAS result, two SNP markers, Cse_sc016995.1_21245 and Cse_sc001760.1_56613, were selected from each linkage group according to p-value to develop ASP-PCR markers. The genotyping assay showed that genotypes of Cse_sc001760.1_56613 and Cse_sc016995.1_21245 in ‘Ariesu’ were heterozygotes of T/C (Y) and A/G (R), respectively, while those in ‘Yellow Queen’ were homozygotes T/T (T) and A/A (A). In the F_1_ population, segregation data for each SNP, Cse_sc001760.1_56613 and Cse_sc016995.1_21245, was heterozygotes:homozygotes = 51:51 and 54:48, respectively (Fig. 3), fitting to the 1:1 segregation ratio for simplex × nulliplex (1 × 0) for hexasomic inheritance. Subsequently, when the two genotypes were combined, genotype data completely explained differences in carotenoid concentrations (Fig. 3); F_1_ plants carrying at least one of the SNPs identical to either C or G exhibited low carotenoid concentration, whereas plants homozygous for the ‘Yellow Queen’ alleles accumulated carotenoids to high concentrations in their petals of ray florets. Segregation data for combined SNP genotypes were YR:TR:YA:TA = 24:30:27:21. This ratio was a good fit to the 2:3:3:2 ratio (χ^2^ = 1.09, *P* = 0.78) for hexasomic duplex × nulliplex, thus indicating that two SNPs had no linkage. This observation, as well as the genetic map position, supported the model that the two loci were genetically identical, being the hexasomic duplex × nulliplex cross.

**Figure 4:**
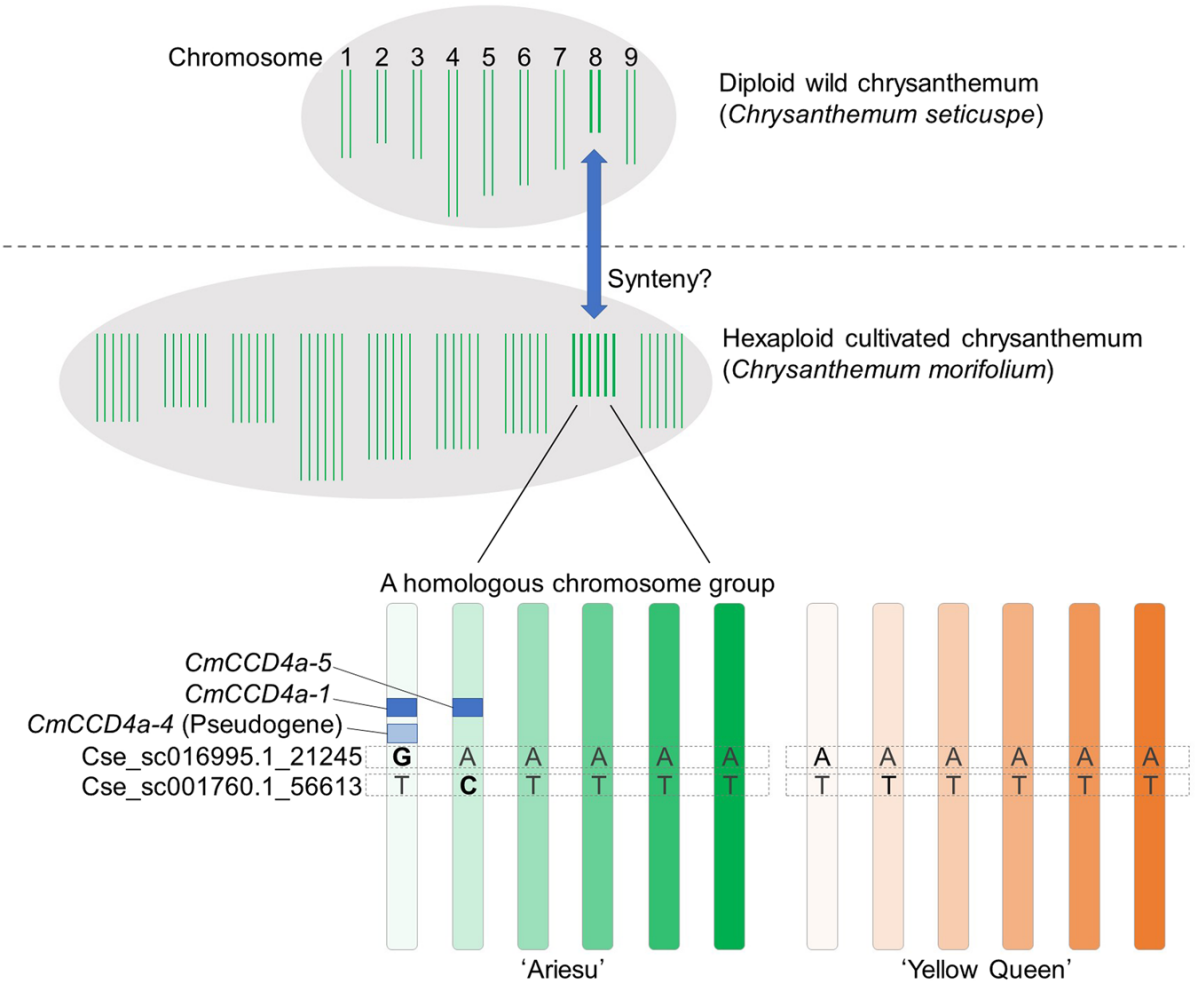
Hypothetical positions of *CmCCD4a* and single-nucleotide polymorphism markers on the six homologous chromosomes of ‘Ariesu’ and ‘Yellow Queen’

Finally, genotypes of the two SNPs and the two genes, *CmCCD4a-1 and CmCCD4a-5*, were associated. The G allele of Cse_sc016995.1_21245 SNP and presence of *CmCCD4a-1* were found to be completely in the coupling phase. Furthermore, the C allele of Cse_sc001760.1_56613 SNP and the presence of *CmCCD4a-5* were also found to be in the coupling phase. No recombination between the SNPs and *CmCCD4a* was observed in the F_1_ population (see Supplementary Table S1). The results indicated that the two functional alleles of *CmCCD4* were completely and separately tagged with SNPs. Here, we propose a model indicating the states of the *CmCCD4* locus in the hexaploid genome for ‘Ariesu’ and ‘Yellow Queen’ (Fig. 4).

## Discussion

The complexity of inheritance patterns resulting from autohexaploidy, large chromosome number and self-incompatibility inhibits the efficient development of DNA markers in chrysanthemum. In this study, we showed that, using simplex SNP markers, GWAS circumvents the problems and enables the development of DNA markers The technical scheme is as follows: (1) double digest restriction site-associated DNA (dd-RAD-Seq) of both parents and the F_1_ population, (2) SNP calling by mapping onto the reference genome sequence of diploid *C*. *seticuspe*, (3) selection of simplex and double-simplex SNP loci based on allele frequency across the population and (4) genotype–phenotype association, using GWAS. In addition, we used biparental populations for the GWAS, in which the SNP allele frequency could be predicted. Dominant alleles at simplex and double-simplex SNP loci would be observed in 50% and 75% of the progeny, respectively. It is possible to select high confident (double-)simplex SNPs supported with low read coverage with a robust filtering conditions to avoid errors^8^. Furthermore, while the numbers of simplex and double-simplex SNPs are limited, the SNPs may be sufficient to trace chromosome recombination breakpoints in a biparental population because of the limitation of the recombination.

We applied this set of methods to carotenoid concentrations in petals of ray florets. For this character, the inhibition of carotenoid formation is dominant, with inheritance at one genetic locus in a hexasomic pattern derived from random chromosome pairing being reported^11^ and the causal gene *CmCCD4a* being identified^15^. ‘Ariesu’, a parent used in this study, was duplex for the carotenoid cleavage allele. As a result, SNP markers were developed for each allele and perfectly corresponded to both carotenoid cleavage in petals of ray florets and the presence or absence of the causal genes *CmCCD4a-1* and *CmCCD4a-5* in each individual of the F_1_ population (see Supplementary Table S1). This clearly verifies the effectiveness of the GWAS method.

Cultivated chrysanthemum lags behind other important plant species in terms of the development of DNA marker because of its genome complexity—arising from the large genome size—and high genome diversity, as well as its autopolyploidy. Two important strategies to overcome the problems of genome complexity are to generate highly reproducible polymorphism information and to circumvent a complex inheritance pattern. dd-RAD-Seq, based on next-generation sequencing technology, enabled the comprehensive and efficient analysis of polymorphisms in the large genome of chrysanthemum. dd-RAD-Seq can read important regions of a genome using restriction enzymes to reduce genome complexity and to avoid repetitive regions of the genome, which make up >70% of the chrysanthemum genome^18^. High genome diversity in chrysanthemum, arising from autopolyploidy and heterozygosity as a result of self-incompatibility, can achieve very high levels of polymorphism. Hirakawa *et al*.^18^ reported that nearly 1 million SNPs were identified among six chrysanthemum varieties, and approximately 52,000 SNPs were identified in the present study. However, we aimed to increase reproducibility using only 9,219 of the single-dose (simplex and double-simplex) SNP markers. Position information of these markers is strongly stable even in autohexaploids because they link to only one allele at one genetic locus on one chromosome. If non-single-dose SNP markers, such as duplex, triplex, or multi-locus markers, were used, then many false-positive SNP markers would be detected by GWAS, with reproducibility in PCR experiments suffering because of doubt concerning their location in the large and complex genome of autohexaploid chrysanthemum.

QTL analysis, a powerful tool to develop DNA markers in crops, is little used in autohexaploids^9,19^. Estimation of recombination frequency requires complicated calculations in QTL analysis in autohexaploids because of the complexity of inheritance patterns due to random chromosome pairing. In contrast, the GWAS approach was designed to detect associations between DNA markers and phenotypes based on the linkage disequilibrium^20^, whereas linkage disequilibrium is actually broken down by recombination. In the present study, we used an F_1_ population experiencing recombination following crossing between two parents. However, recombination frequency is usually very low. Therefore, ignoring recombination did not matter in the results. In conclusion, GWAS, using single-dose SNP markers, is a simple method to handle a complex inheritance pattern for the development of DNA markers in autohexaploids and can be also applicable to other autopolyploid plants with genome complexity, such as sweet potato and sugarcane.

In the present study, the genome sequence of the wild diploid chrysanthemum *C*. *seticuspe* was used as a reference for mining SNP markers from dd-RAD-Seq data from autohexaploid cultivated chrysanthemum, by assuming synteny of genome sequences between these two species, although *C*. *seticuspe* is generally not thought to be a direct progenitor of the cultivated chrysanthemum^21,22^. Wild chrysanthemums are divided into yellow- and white-flowered groups, according to whether the species carried functional *CCD4a* responsible for carotenoid cleavage in petals of ray florets^15^. *C*. *seticuspe* belongs to a yellow-flowered group, so that this species is considered to have no functional *CCD4a* in its genome. Recent genome sequence data support the absence of *CCD4a* in the two yellow-flowered wild chrysanthemum species *C*. *seticuspe*^18^ and *C*. *nakingense*^23^. However, here, SNP markers for carotenoid cleavage in petals of ray florets were consequently developed. Moreover, SNP markers were located relatively close to *CmCCD4a* on the genome of cultivated chrysanthemum. This shows that *C*. *seticuspe* has genome regions corresponding to the peripheral genome region of *CCD4a* in cultivated chrysanthemum, although *C*. *seticuspe* does not itself carry *CCD4a* on its genome. This information and the fact that carotenoid cleavage is the dominant character suggest that yellow-flowered wild chrysanthemums have developed from white-flowered species by the loss of *CCD4a*. *Chrysanthemum* species range from diploid to decaploid. In the present study, the diploid *C*. *seticuspe* genome proved to be applicable to genetic analyses in hexaploid cultivated chrysanthemums, suggesting a generally conserved synteny among the genomes of *Chrysanthemum* species.

We measured carotenoid concentrations in each F_1_ individual. Concentration values were clearly segregated into two groups depending on whether carotenoid cleavage had occurred (Fig. 1). The distribution of carotenoid concentrations in 21 carotenoid-accumulated F_1_ individuals, which were absence of *CmCCD4a* gene, ranged from 29.6 μg·g^−1^ FW to 130.4 μg·g^−1^ FW (Fig. 1 and see Supplementary Table S1), being roughly <50% the concentration found in the parent ‘Yellow Queen’ (216.9 μg·g^−1^ FW). Petal carotenoid concentrations are quantitative traits determined by multiple mechanisms involving several factors and genes^24^. This distribution also genetically indicated that the level of carotenoid concentration in a chrysanthemum plant carrying no functional *CmCCD4a* is a quantitative trait, thus confirming the qualitative and quantitative control of carotenoid concentration of petals in chrysanthemums. Carotenoid cleavage via *CCD4a* preferentially determines whether carotenoid is accumulated in petal, similar to that reported in previous studies^15,17^. Without functional *CCD4a*, carotenoid is accumulated and the level can be quantitatively regulated. We tried to develop SNP markers associated with this quantitative trait of carotenoid accumulation, but no markers showed significant association with a quantitative locus in terms of carotenoid concentration in petals of ray florets by GWAS using simplex markers. This is an inevitable result because the number of F_1_ individuals accumulating carotenoids in petals of ray florets was too small for analysis.

In the case of quantitative traits in an autohexaploid, we need to pay greater consideration to the dosage effect by allele dosage such as nulliplex, simplex, duplex, triplex and so on as well as to multiple loci. When we discuss the structure of heterozygous genotypes on a locus with two alleles in an autohexaploid, there are five different combinations of two alleles, ranging from one to five copies (Aaaaaa–AAAAAa). Adding two homozygous genotypes (AAAAAA and aaaaaa) means that a total of seven different pattern of allele dosage can exist at a single locus. Using only simplex markers, it is challenging to determine whether GWAS can detect such dosage effects in quantitative traits, although duplex inheritance, involving two alleles that qualitatively and redundantly determine carotenoid cleavage in petals of ray florets, could be detected in this study. We need to be able to handle duplex and triplex markers, which were not used in the present study because of the lack of sufficient genotyping accuracy, in GWAS to develop DNA markers of quantitative traits in polyploid plants.

## Methods

### Plant materials and DNA extraction

A pair of F_1_ populations originated from reciprocal crosses between the pink-flowered cultivar, ‘Ariesu’ (obtained from the National Federation of Agricultural Cooperative Associations, Japan), due to extremely low level of carotenoid pigment (but the presence of anthocyanin pigment) in petals of ray florets, and a yellow-flowered cultivar, ‘Yellow Queen’ (obtained from Genebank Project, NARO, Japan), containing carotenoid in the petals of ray florets. A total of 61 seedlings from ‘Ariesu’ × ‘Yellow Queen’ (namely AY population) and 41 from ‘Yellow Queen’ × ‘Ariesu’ (YA) were planted in plastic pots (12-cm internal diameter, one seedling per pot) containing a commercial horticultural soil (Kureha-Engei-Baido; Kureha Chemical Co. Ltd., Tochigi, Japan) and maintained in the vegetative state in a glasshouse (heated when the temperature dropped <18 °C and ventilated when it rose >25 °C) under 6-h night-break conditions. Genomic DNA was extracted from the shoot tips (30 mg fresh weight) using the DNeasy Plant Mini Kit (Qiagen, Hilden, Germany) according to the manufacturer’s instructions.

### Phenotyping of carotenoid concentrations

Rooted cuttings of the 102 F_1_ individuals from the AY and YA populations and the two parents were planted in plastic pots (9-cm internal diameter, one cutting per pot) containing a commercial horticultural soil (Kureha-Engei-Baido; Kureha Chemical Co. Ltd., Tochigi, Japan) and grown to flowers in May 2016 under a 11.5-h photoperiod using black-out curtains in a glasshouse at NARO (Tsukuba, Japan). Petals of ray florets (50 mg fresh weight) from the terminal capitulum were collected at anthesis. Each sample was ground in 100 μl acetone in a 1.5-ml microtube. Subsequently, 700 μl diethyl ether and 50 μl ultrapure water were added. The microtube was shaken using a vortex mixer and centrifuged at 14,000 × g for 5 min. The diethyl ether layer was collected and decanted into a new microtube, where it was dried, dissolved in 100 μl *N*, *N*-dimethylformamide and used in quantitative analyses.

The absorbance of the solution (in a 2 μl aliquot) was measured using a NanoDrop 1000 spectrophotometer (Thermo-Fisher Scientific K. K., Yokohama, Japan) at wavelengths between 350 nm and 600 nm. The total concentration of carotenoids was calculated from the absorbance at the 450 nm absorption maximum using the E^1%^ value of lutein (2550)^25^, which was defined as the theoretical absorbance of a 1% solution in a cell of 1-cm pathlength and expressed as micrograms of lutein equivalent per gram fresh weight (g^−1^ FW) of tissue. Measurements were performed in duplicates for each individual.

### Cloning and genotyping of carotenoid cleavage genes (*CmCCD4a*)

Primers were designed in the conserved region of six *CmCCD4a* homologs (see Supplementary Table S2), *CmCCD4a-1* (GenBank accession number: AB627797), *CmCCD4a-2* (AB627798), *CmCCD4a-3* (AB627800), *CmCCD4a-4* (AB627802), *CmCCD4a-5* (AB695091) and *CmCCD4a-6* (AB695092), and used for the genomic PCR using 5 ng of genomic DNA from ‘Ariesu’ or ‘Yellow Queen’ as templates with KOD-Plus-Neo DNA polymerase (Toyobo, Osaka, Japan) under conditions of 98 °C for 30 s, followed by 30 cycles of 98 °C for 10 s and 68 °C for 2 min, and a final extension at 72 °C for 5 min. PCR products (2 μl/sample) were electrophoresed on an 0.8% agarose gel, visualized after ethidium bromide staining under UV light using a gel imaging instrument (Gel Doc EZ System, Bio-Rad, Tokyo, Japan) and extracted from gels using QIAquick PCR & Gel Cleanup Kit (Qiagen, Hilden, Germany).

Deoxyadenosine monophosphate was added to the 3′ end of the PCR products with the 10 × A-attachment mix (Toyobo, Osaka, Japan). Products were cloned into the pGEM-T Easy Vector (Promega, Madison, WI, U.S.A.) and sequenced with a ABI-3500 capillary sequencer based on the Sanger method. Sequence data were analyzed using BioEdit software^26^.

Genotypes of *CmCCD4a-1*, *CmCCD4a-4* and *CmCCD4a-5* in each F_1_ plant were analyzed by PCR using 10 ng of genomic DNA. Specific primers were designed corresponding to each *CmCCD4a* (see Supplementary Table S2). PCR were performed using each primer pair under conditions of 95 °C for 30 s, 30 cycles of 95 °C for 5 s and 60 °C for 30 s, with a SYBR Premix Ex Taq II Tli RNase H plus (TaKaRa Bio, Shiga, Japan) on a thermal Cycler Dice Real-Time system (TaKaRa Bio, Shiga, Japan). Each *CmCCD4a* showed threshold cycle number response at 24–27 cycles.

### dd-RAD-Seq analysis

Genomic DNA from the F_1_ population and its parental lines was double-digested with *Pst*I and *Msp*I to generate dd-RAD-Seq library, as described in Shirasawa *et al*.^27^. Nucleotide sequences of the libraries were determined on a HiSeq. 2000 (Illumina) platform in paired-end, 93-bp mode and a MiSeq (Illumina) platform in paired-end, 251-bp mode.

### Data processing

Primary data processing of sequence reads was performed as described by Shirasawa *et al*.^8^, with minor modifications. Low-quality sequences with quality scores of <10 were removed from the 3′ ends using PRINSEQ (version 0.20.4)^28^, and adapters were trimmed with fastx_clipper in FASTX-Toolkit (version 0.0.13) (http://hannonlab.cshl.edu/fastx_toolkit). The remaining high-quality reads were mapped onto the *C*. *seticuspe* genome sequence (CSE_r1.0)^18^ as a reference using Bowtie 2 (version 2.2.3)^29^ with parameters of maximum fragment size length, 1000 (I = 1000), in the ‘–sensitive’ preset of the ‘–end-to-end’ mode. The resultant sequence alignment/map format (SAM) files were converted to binary sequence alignment/map format (BAM) files and subjected to SNP calling using the mpileup option of SAMtools (version 0.1.19)^30^ and the mpileup2snp option of VarScan 2 (version 2.3)^31^ to obtain a variant call format (VCF) file including SNP information.

### SNP mining

In diploid species, the use of read counts of pooled progeny’s samples at each SNP locus to estimate the genotypes of the parental lines has been suggessted^32^. This approach is also effective in hexaploid species^8^, whereas seven genotypes (nulliplex to hexaplex) should be estimated. High-confidence SNP candidates were selected using VCFtools (version 0.1.12b)^33^ with the following criteria: (i) depth of coverage ≥10 for each data point and (ii) proportion of missing data <0.25 for each locus. Using the filtered VCF files, AAF for each position was calculated as described in Shirasawa *et al*.^8^ with the following formula:

$$({\rm{Number}}\,{\rm{of}}\,{\rm{reads}}\,{\rm{with}}\,{\rm{variant}} \mbox{-} {\rm{supporting}}\,{\rm{bases}})/({\rm{Number}}\,{\rm{of}}\,{\rm{total}}\,{\rm{reads}}\,{\rm{aligned}}\,{\rm{at}}\,{\rm{the}}\,{\rm{position}}).$$

Double-simplex SNP sites of “AAAAAa × AAAAAa (AAF = 2/12 = 0.1667)” and “Aaaaaa × Aaaaaa (AAF = 10/12 = 0.8333)” were selected with an AAF ≥ 0.1250 and <0.2083 and those with an AAF ≥ 0.7917 and <0.8750, respectively. In addition, simplex SNP sites of “AAAAAA × AAAAAa (AAF = 1/12 = 0.0833)” and “Aaaaaa × aaaaaa (AFF = 11/12 = 0.9167)” were selected with an AAF ≥ 0.0417 and <0.1250 and those with an AAF ≥ 0.8750 and <0.9583, respectively. Furthermore, on the basis of genotypes of individuals of the SNP loci, the genotype of each individual determined. Theoretically, the “AAAAAa × AAAAAa” double-simplex SNPs, for example, would be expected to segregate into AAAAAA (AAF = 0/6 = 0.000), AAAAAa (AAF = 1/6 = 0.167) and AAAAaa (AAF = 2/6 = 0.333) at a ratio of 1:2:1 in the F_1_ progeny. However, as mentioned in Shirasawa *et al*.^8^, it was difficult to distinguish between the AAAAAa and AAAAaa genotypes because the numbers of reads of each individual were insufficient to significantly differentiate between AAFs of 0.167 and 0.333. Therefore, AAFs of 0 and >0.000 were scored as homozygous (0/0) and non-homozygous REF alleles (0/1), respectively, with an expected segregation ratio of 1:3, as with dominant loci. Correspondingly, AAFs of 1 and <1.000 were encoded as homozygous (1/1) and non-homozygous ALT alleles (0/1), respectively, for following GWAS. This limitation made it impossible to score intermediate AAF values from duplex SNPs “AAAAaa × AAAAaa” and triplex “AAAaaa × AAAaaa” because of insufficient genotyping accuracy. Furthermore, subsets of segregation data of double-simplex and simplex loci fitting the expected ratio of 3:1 and 1:1, respectively, as assessed by chi-squared tests (*P* ≥ 0.01), were selected.

### Genome-wide association study

Associations between genotypes and phenotypes were analyzed with the general linear model using the TASSEL program^34^ with the default parameters. The thresholds for the association were set as 5.4 × 10^−6^ (=0.05/9219) at a significant level of 5% after Bonferroni multiple test correction^35^.

### Validation of SNPs associated with carotenoid cleavage

SNP markers significantly associated with carotenoid cleavage in petals of ray florets were validated using the ASP-PCR analysis using 10 ng of genomic DNA of the parents and F_1_ individuals. Specific primers corresponding to SNPs were designed so that their 3′ terminal nucleotides perfectly corresponded with the SNP match with one allele (the specific allele) but had a 3′ mismatch with other alleles (the non-specific alleles) (see Supplementary Table S2). ASP-PCR were performed using each primer pair under conditions of 95 °C for 30 s, 30 cycles of 95 °C for 5 s and 60 °C for 30 s, with a SYBR Premix Ex Taq II Tli RNase H plus (TaKaRa Bio, Shiga, Japan) on a thermal Cycler Dice Real-Time system (TaKaRa Bio, Shiga, Japan). Each SNP showed threshold cycle number response at 24–28 cycles.

## Supplementary information

Supplementary-Information

## Data Availability

The sequence reads were registered in Sequence Read Archive database in DNA Data Bank of Japan (accession number DRA007925).
