# Supplementary material for: Genome-wide association study overcomes the genome complexity in autohexaploid chrysanthemum and tags SNP markers onto the flower color genes
Source: Sci Rep. 2019 Sep 26;9:13947. doi: 10.1038/s41598-019-50028-z (PMC6763435; doi:10.1038/s41598-019-50028-z)
Supplement: Supplementary file 1 — Supplementary-Information [file 41598_2019_50028_MOESM1_ESM.pdf]

Supplementary Information

Genome-wide association study overcomes the genome complexity in autohexaploid chrysanthemum and tags SNP markers onto the flower color genes

Katsuhiko Sumitomo, Kenta Shirasawa, Sachiko Isobe, Hideki Hirakawa, Tamotsu Hisamatsu, Yoshihiro Nakano, Masafumi Yagi, Akemi Ohmiya

Supplementary Table S1. Phenotype of carotenoid cleavage in petals of ray florets and genotypes of *CmCCD4a* genes and SNP alleles by ASP-PCR in F_1 population.

Plants of F_1 population	Carotenoid cleavage	*CmCCD4a*			G allele on Cse_sc016995.1_21245	C allele on Cse_sc001760.1_56613
		-1	*-4*	*-5*		
AY-01	−	−	−	−	−	−
AY-02	−	−	−	−	−	−
AY-03	+	+	+	−	+	−
AY-04	−	−	−	−	−	−
AY-05	+	−	−	+	−	+
AY-06	+	+	+	−	+	−
AY-07	+	−	−	+	−	+
AY-09	+	−	−	+	−	+
AY-10	+	+	+	+	+	+
AY-11	+	+	+	+	+	+
AY-12	+	−	−	+	−	+
AY-13	+	+	+	−	+	−
AY-14	+	+	+	+	+	+
AY-15	+	−	−	+	−	+
AY-16	+	−	−	+	−	+
AY-17	−	−	−	−	−	−
AY-18	−	−	−	−	−	−
AY-19	+	−	−	+	−	+
AY-20	+	+	+	−	+	−
AY-21	+	+	+	−	+	−
AY-22	−	−	−	−	−	−
AY-23	+	+	+	+	+	+
AY-24	+	+	+	+	+	+
AY-25	+	−	−	+	−	+
AY-26	−	−	−	−	−	−
AY-27	+	−	−	+	−	+
AY-29	+	+	+	+	+	+
AY-30	−	−	−	−	−	−
AY-31	+	+	+	+	+	+
AY-32	−	−	−	−	−	−
AY-33	+	+	+	+	+	+
AY-34	+	+	+	+	+	+
AY-35	+	+	+	−	+	−
AY-36	+	+	+	+	+	+

AY-37	+	+	+	−	+	−
AY-38	−	−	−	−	−	−
AY-39	+	+	+	+	+	+
AY-40	−	−	−	−	−	−
AY-41	+	+	+	−	+	−
AY-43	+	−	−	+	−	+
AY-44	+	+	+	−	+	−
AY-45	+	+	+	+	+	+
AY-46	−	−	−	−	−	−
AY-47	+	+	+	+	+	+
AY-48	+	+	+	+	+	+
AY-49	+	−	−	+	−	+
AY-50	+	+	+	−	+	−
AY-51	+	+	+	+	+	+
AY-52	+	−	−	+	−	+
AY-53	+	+	+	−	+	−
AY-54	+	+	+	−	+	−
AY-55	+	+	+	+	+	+
AY-56	+	+	+	−	+	−
AY-57	+	+	+	+	+	+
AY-58	+	+	+	−	+	−
AY-59	+	+	+	−	+	−
AY-60	+	+	+	+	+	+
AY-61	+	+	+	−	+	−
AY-63	+	+	+	−	+	−
AY-64	+	−	−	+	−	+
AY-65	+	+	+	+	+	+
YA-01	+	−	−	+	−	+
YA-02	+	−	−	+	−	+
YA-04	+	−	−	+	−	+
YA-05	+	−	−	+	−	+
YA-06	−	−	−	−	−	−
YA-07	+	−	−	+	−	+
YA-08	−	−	−	−	−	−
YA-09	−	−	−	−	−	−
YA-10	+	−	−	+	−	+
YA-11	+	+	+	+	+	+
YA-12	+	+	+	−	+	−

YA-13	+	+	+	−	+	−
YA-14	+	−	−	+	−	+
YA-15	−	−	−	−	−	−
YA-16	+	+	+	+	+	+
YA-17	+	−	−	+	−	+
YA-18	+	+	+	+	+	+
YA-19	+	+	+	−	+	−
YA-20	+	+	+	+	+	+
YA-21	+	−	−	+	−	+
YA-22	+	+	+	−	+	−
YA-23	+	+	+	−	+	−
YA-24	+	+	+	−	+	−
YA-25	+	+	+	−	+	−
YA-26	−	−	−	−	−	−
YA-27	+	−	−	+	−	+
YA-28	−	−	−	−	−	−
YA-29	−	−	−	−	−	−
YA-30	+	−	−	+	−	+
YA-40	+	+	+	−	+	−
YA-41	+	+	+	−	+	−
YA-42	−	−	−	−	−	−
YA-43	+	+	+	−	+	−
YA-44	+	+	+	−	+	−
YA-45	+	−	−	+	−	+
YA-46	−	−	−	−	−	−
YA-47	+	+	+	+	+	+
YA-49	+	+	+	−	+	−
YA-50	+	−	−	+	−	+
YA-51	+			+		+
YA-52	+	+	+	−	+	−

+: presence

－: absence

Supplementary Table S2. Primers used in this study.

Target	Sequence (5′ -> 3′)	
	Forward	Reverse
Conserved region of six *CmCCD4a* homologs	CATCCCTTACCAAATTATGGGCTC	GAAAGCTGTGTACTCAACGTCAATA
CmCCD4a-1 (AB627797)	GGGCGTGGTTGTTTTGGAGGCGAA	TGCACATAAGAAACTATGTAACC
CmCCD4a-4 (AB627802)	ATCGCGAGTGTATTGTGGCGAT	TGCACATAAGAAACTATGTAACC
CmCCD4a-5 (AB695091)	GGGCATGGTTGTTTTGGCGGTGAG	TGCACATAAGAAACTATGTAACC
CmFTL3 (AB770479) for PCR positive control	AACGGGTGTGAGCTAAAACC	TGGAGCATCAGGATCTACC
A allele on Cse_sc016995.1_21245	AATCATACTCTCTGCAGGTG	ATGCCACTCAACATCAAACGT
G allele on Cse_sc016995.1_21245	AATCATACTCTCTGCAGGTG	ATGCCACTCAACATCAAACGC
T allele on Cse_sc001760.1_56613	TGGCTTAACCGGGGTGGCTT	AGAAACACAAATGCCTCATG
C allele on Cse_sc001760.1_56613	TGGCTTAACCGGGGTGGCTC	AGAAACACAAATGCCTCATG

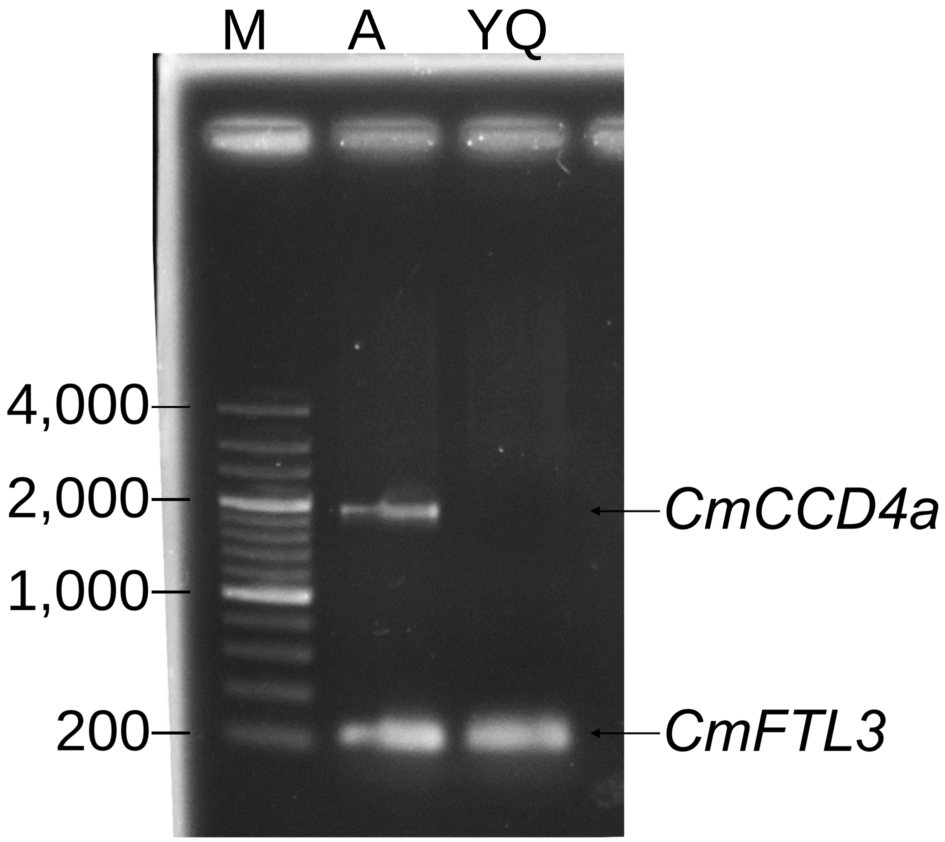

Supplementary Figure S1. Genomic PCR analysis of *CmCCD4a* in óAriesuô(A) and óYellow Queenô(YQ).

A *CmFTL3* (DDBJ accession number: AB770479) primer set was used as a PCR control. M: molecular mass marker in bp.

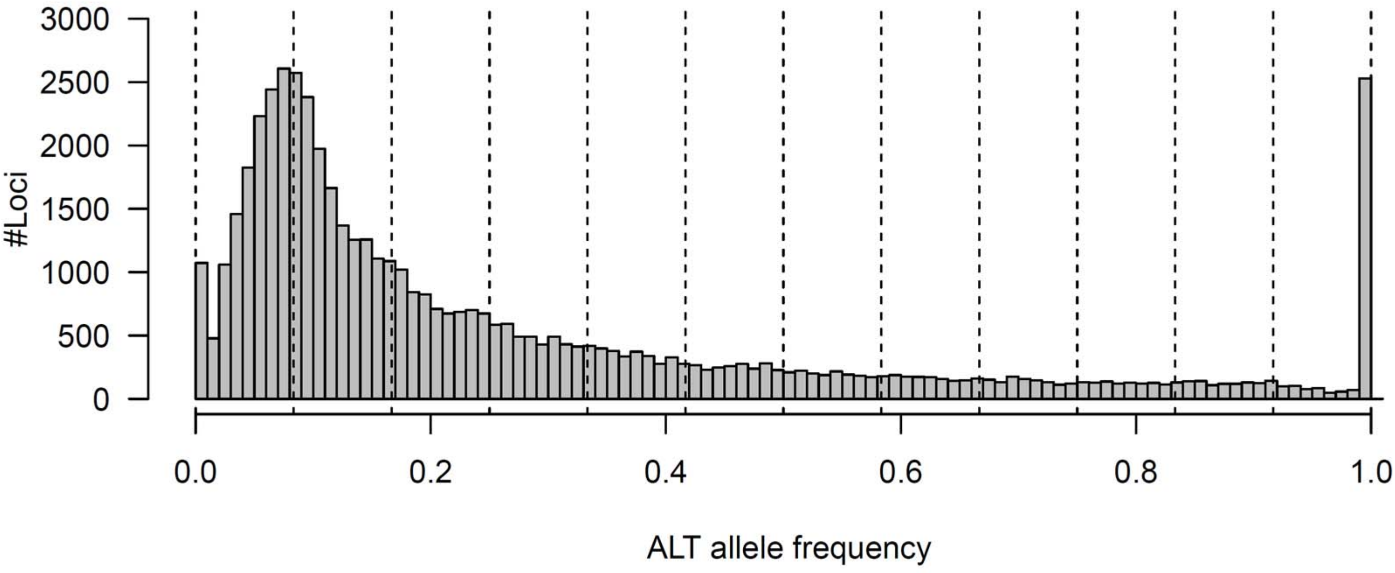

Supplementary Figure S2. Distribution of ALT allele frequency in the F_1 mapping population derived from a cross between 'Ariesu' and 'Yellow Queen'.
Vertical dashed lines indicate frequencies of 0.0000, 0.0833, 0.1667, 0.2500, 0.3333, 0.4167, 0.5000, 0.5833, 0.6667, 0.7500, 0.8333, 0.9167 and 1.0000.

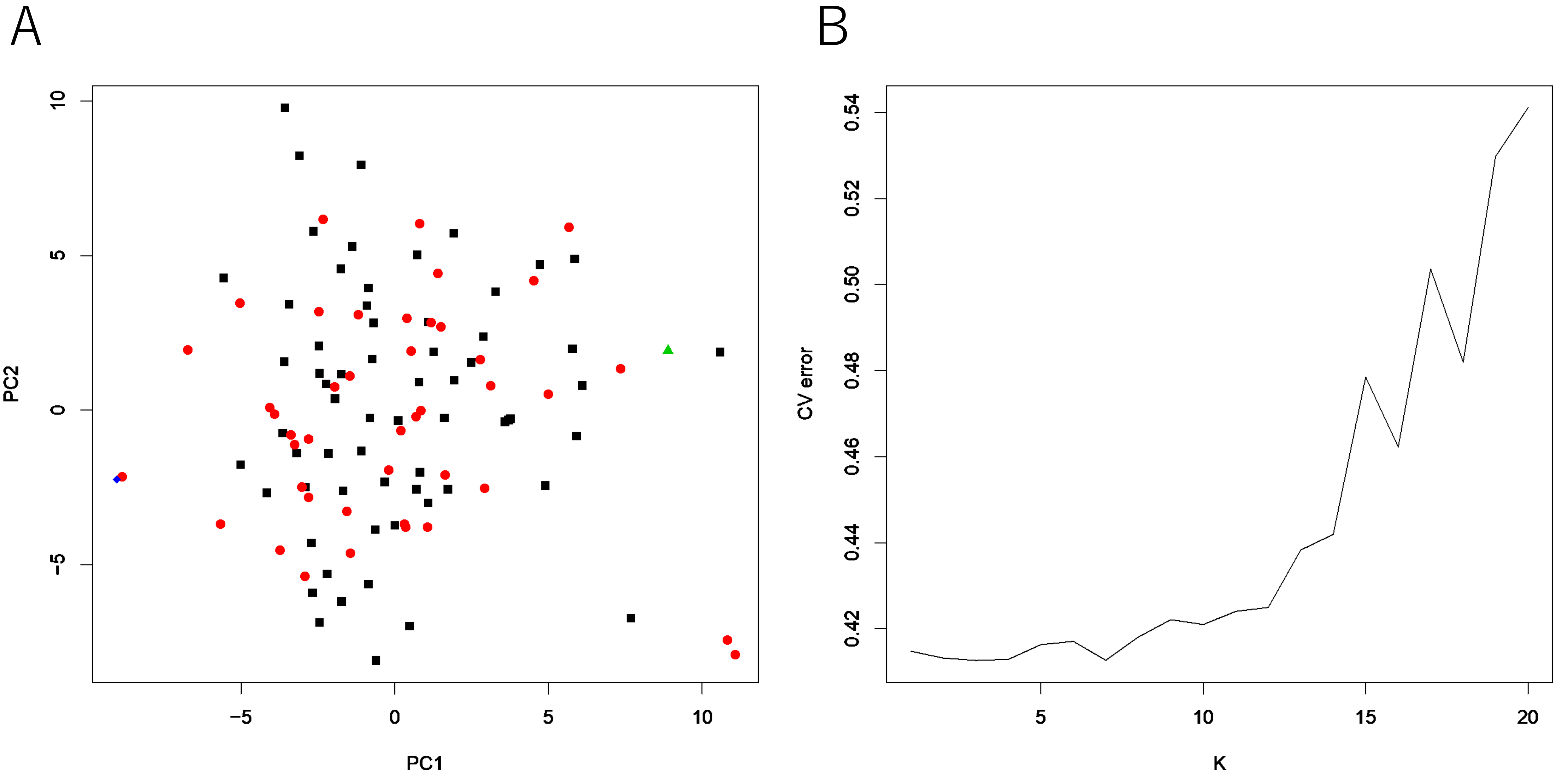

Supplementary Figure S3. Structure of F_1 mapping population

A. Principal component analysis based on SNP genotyping data for the population. 'Ariesu', 'Yellow Queen', and the reciprocal F_1 populations, AY and YA, are indicated by green, blue, black, and red dots, respectively. **B.** Cross-validation errors for each cluster number in Admixture analysis.
